# Subtype-specific atypical B cell profiles in myasthenia gravis reveal distinct immunopathological pathways

**DOI:** 10.3389/fimmu.2025.1608160

**Published:** 2025-06-18

**Authors:** Patricia M. Sikorski, Henry J. Kaminski, Angela Vincent, Taylor Bauman, Leslie Jacobson, Linda L. Kusner

**Affiliations:** ^1^ Department of Neurology & Rehabilitation Medicine, George Washington University, Washington, DC, United States; ^2^ Neurosciences Group, Department of Clinical Neurology, Weatherall Institute of Molecular Medicine, John Radcliffe Hospital, Oxford, United Kingdom; ^3^ Department of Pharmacology & Physiology, George Washington University, Washington, DC, United States

**Keywords:** myasthenia gravis, acetylcholine receptor, muscle-specific tyrosine kinase, atypical B cells, autoimmunity

## Abstract

**Introduction:**

Atypical B cell (atBC) subsets display significant heterogeneity across autoimmune diseases, complicating efforts to define their role and therapeutic potential. We hypothesized that this heterogeneity reflects the responses to specific immunopathology, resulting in disease-specific profiles. The myasthenia gravis (MG) subtypes acetylcholine receptor (AChR)-positive MG and muscle-specific kinase (MuSK)-positive MG provide an ideal model to explore atBCs due to the distinct immune mechanisms driven by IgG1-3 and IgG4 autoantibodies, respectively in the disease.

**Methods:**

CD11c^+^ and IgD^−^CD27^−^ double-negative (DN) B cells were analyzed by spectral flow cytometry in non-autoimmune controls, AChR-MG, and MuSK-MG. Results were correlated with clinical parameters and antibody levels. In MG subtypes, atBC subsets were further examined for the impact of disease onset and prior rituximab treatment. CD11c^+^ B cells were stimulated *in vitro* to assess antibody secreting cell (ASC) differentiation.

**Results:**

CD11c^+^ and DN2 B cells were increased in late-onset AChR-MG, while MuSK-MG featured expanded DN3 B cells linked to disease severity. CD20 expression in atBCs was differentially expressed between MG subtypes, with higher levels in late-onset AChR-MG and significantly reduced levels in MuSK-MG. CD11c^+^ B cells were reduced after anti-CD20 treatment in MuSK-MG, whereas DN B cells were unaffected. Functionally, CD11c^+^ B cells from MuSK-MG exhibited greater ASC differentiation and autoantibody production.

**Discussion:**

MG subtypes exhibit distinct atBC profiles linked to immunopathology and disease onset. These findings reveal subtype-specific pathways that regulate atBCs and highlight their potential as therapeutic targets in both IgG1-3- and IgG4-mediated autoimmunity.

## 1 Introduction

Atypical B cells (atBCs) have gained attention due to their association with autoantibody production and disease manifestations in autoimmune disorders, making them an attractive therapeutic target (1, 2). These cells are distinguished by a protein expression profile that differentiates them from canonical B cells, including increased expression of CD11c, T-bet, and CD20, alongside downregulation of CD21, CXCR5, IgD, and CD27 (3, 4). This has led to the identification of various atBC subsets, including CD11c^+^, IgD^−^CD27^−^ double-negative (DN), and CD21^−^ B cells (1, 5, 6). However, it remains unclear whether this heterogeneity reflects distinct subsets with specialized roles or arises from diverse immune microenvironments.

Among atBC subsets, DN B cells have emerged as particularly relevant to autoimmune disease, with two subsets — DN2 (CXCR5^−^CD11c^+^) and DN3 (CXCR5^−^CD11c^−^) B cells — most closely linked to autoimmunity. DN2 B cells, characterized by CD11c and T-bet expression, are enriched in IgG1-3-driven diseases like systemic lupus erythematosus (SLE), where they correlate with antibody levels and disease severity (7–10). In seropositive rheumatoid arthritis (RA), DN2 B cells are similarly enriched in both blood and synovial fluid and are linked to joint damage and autoantibody production (11, 12). In contrast, DN3 B cells are more closely associated with IgG4-mediated diseases, where they are found both in circulation and infiltrating affected tissues, correlating with plasmablast frequencies (13, 14). While DN3 B cells share some features with DN2 B cells, particularly their association with extrafollicular immune responses (13, 15); their precise role in autoimmunity, particularly in IgG4-driven diseases, remains poorly understood.

Functionally, specific subsets of atBCs can differentiate into ASCs and secrete antibodies. These cells possess unique activation requirements for proliferating and differentiation into ASCs, requiring a synergy of innate and T cell signals (8, 9). Unlike conventional B cells, atBCs do not readily differentiate or proliferate in response to BCR signaling alone. Instead, their activation appears to rely on both external stimuli and internal regulatory pathways. ASC differentiation in atBCs requires a combination of TLR signaling, IFN-γ, and T cell-derived cytokines such as IL-2, IL-21 (8, 16–18) suggesting a specific microenvironment for antibody production. These findings suggest that variations in TLR, cytokine, and BCR signaling may contribute to the functional heterogeneity of atBCs across autoimmune diseases.

Myasthenia gravis (MG) provides a fitting model for investigating atBC heterogeneity, with its distinct subtypes allowing for direct comparison of atBCs in relation to autoantigen-specific immune responses. MG is an autoantibody-driven disease characterized by muscle weakness and two major subtypes: acetylcholine receptor (AChR)-positive MG and muscle-specific kinase (MuSK)-positive MG (19). These subtypes differ significantly in their pathogenic mechanisms and immune profiles. AChR-MG is predominantly mediated by complement-fixing IgG1–3 antibodies, while MuSK-MG is driven by IgG4 autoantibodies that interfere with receptor function. AChR-MG is linked to long-lived plasma cells that sustain antibody production, whereas MuSK-MG relies on short-lived plasmablasts (20). These differences are thought to contribute to variations in response to anti-CD20 therapies, with MuSK-MG typically responding more favorably, likely due to the effective depletion of circulating plasmablasts. AChR-MG can be further classified by age of onset: early-onset MG (EOMG, before age 50) and late-onset MG (LOMG, after age 50), (21). EOMG is closely linked to thymic pathology, where B cell follicles promote localized autoreactive immune responses, while LOMG is associated with loss of T cell tolerance in an aging immune system (19). These distinct immunological features make MG a valuable model for exploring how atBC profiles can shape diverse immune conditions.

In this study, we focused on CD11c^+^ and DN B cells, which are implicated in autoantibody-driven diseases and enriched in various autoimmune conditions. We identified specific atBC subsets associated with autoantibody status and clinical phenotypes in MG and demonstrate CD11c^+^ B cells exhibit differences in ASC differentiation and antibody secretion. We also evaluated the impact of prior rituximab treatment in MuSK-MG to assess the susceptibility of atBC subsets to B cell depletion. Our findings highlight atBCs as distinct subsets associated with MG subtypes, representing a heterogenetic B cell population that vary across autoimmune diseases. These insights may inform strategies to refine targeted therapies in antibody-mediated conditions.

## 2 Materials and methods

### 2.1 Human subjects and ethics statement

Blood samples were collected from patients at the neurology clinics at the George Washington University. Inclusion criteria for MG patients included: 1) clinical diagnosis of generalized or ocular MG, 2) elevated serum AChR or MuSK antibodies 3) immunosuppressant naïve. Inclusion criteria for non-autoimmune control patients included: 1) age and sex matched 2) absence of autoimmune disease. At the time of study, two AChR-MG patients had received prednisone for less than 2 weeks and six of the 19 MuSK-MG patients had been treated with rituximab (18–36 months post treatment) but recovered normal lymphocyte counts. Exclusion criteria applied to both MG and control subjects were active infectious disease and vaccination within 30 days. The Myasthenia Gravis Foundation of America Clinical Classification was used to define disease severity at the time of blood draw. **Table 1** shows demographic and clinical characteristics of the subjects. All participants provided written consent. The study was approved by the George Washington University Institutional Review Board.

**Table 1:** Subject characteristics and demographics for flow cytometry analysis.

Characteristics	Controls (n = 14)	AChR-MG (n = 24)	MuSK-MG (n = 19)
Sex, Female % (n)	71.4 (10)	62.5 (15)	94.7 (18)
Average age, years (range)	46.7 (23-68)	50.84 (18-87)	40.3 (22-64)
Disease duration, months (range)	–	56.43 (1-156)	132.11 (6-528)
Disease onset			
Late onset, n (%)	–	12 (50)	–
Early onset, n (%)	–	12 (50)	–
Disease (MGFA) classification			
Class I (ocular)	–	6 (25.00)	0 (0.0)
Class II (mild)	–	5 (20.83)	10 (52.63)
Class III/IV (moderate/severe)	–	13 (54.17)	7 (36.84)
Remission	–	0 (0)	2 (10.53)
Treatment or medication, no. (%)			
Prednisone	–	2 (8.33)	5 (26.31)
Thymectomy	–	1 (4.17)	1 (5.2)
IVIG	–	1 (4.17)	2 (10.5)
Acetylcholinesterase inhibitor	–	9 (37.50)	0 (0.0)
Mycophenolate	–	0 (0.0)	1 (5.2)
Previous Rituximab	–	0 (0.0)	6 (31.57)
18 months post treatment	–	0 (0.0)	1
24 months post treatment	–	0 (0.0)	2
> 36 months post treatment	–	0 (0.0)	3
No treatment	–	11 (45.83)	6 (31.57)

### 2.2 PBMC isolation

Blood was collected in ACD tubes and PBMCs were isolated using Ficoll density gradient centrifugation. Briefly, blood was transfer to a 50 mL SepMate tube containing 15 mL of Lymphoprep (StemCell Technologies) and centrifuged at 1200 xg for 10 minutes at room temperature. The PBMC layer was collected and washed three times with 45 mL of HBSS. Cells were frozen down in Cyrostor (StemCell Technologies) freezing media (7-10x10^6^ cells per mL of freezing media). After slow cooling to -80 degrees C, cells were transferred to liquid nitrogen for long term storage.

### 2.3 B cell isolation

PBMCs were thawed and washed once with complete IMDM (10% FBS and 1% Pen/Strep) and once with B cell isolation buffer (PBS + 2% FBS + 10 mM EDTA) at 350 x *g* for 10 minutes. PBMCs were resuspended in B cell isolation buffer at a concentration of 5x10^7^/mL and B cells were isolated using the StemCell Technologies B cell Isolation Kit according to the manufacturer’s instructions (Catalog number 17954). Purity was assessed by flow cytometry (**Supplementary Figures 1A, B**).

### 2.4 Flow cytometry

*Antibodies*. All antibodies were purchased from BioLegend unless otherwise stated. The following clones were used in this study: CD3-BV650 (clone UCHT1), CD20-AlexFluor 700 (clone 2H7), CD27-BV605 (clone O323), CD21-PE-Cy7 (clone Bu32), CD11c-PE (clone 3.9), CXCR5-FITC (clone J252D4), CD95-BV421 (clone DX2), IgD-PerCPCy5.5 (clone IA6-2), FCRL5-APC (cline 509f6), CD38-PE-Dazzle594 (clone HB-7) and T-bet-BV711 (clone 4B10) (**Supplementary Table 1**). Titrations of each antibody was performed to determine optimal concentration.

#### 2.4.1 Extracellular staining

Isolated B cells were washed twice with PBS, resuspended in 1 mL of PBS, and stained with Zombie Aqua (BioLegend, Cat. No. 423101) viability dye (1:1000 per 10^6^ cells) for 30 minutes on ice. Cells were washed twice with cold complete IMDM media. Fc receptors were blocked with Human TruStain FcX (BioLegend, Cat. No. 422302) in cold media for 10 minutes on ice. Cells were then plated into a 96 well plate and stained with antibodies in Brilliant Violent Stain buffer (BD) for 30 minutes on ice. Cells were washed twice with 200 µl cell staining buffer (BioLegend, Cat. No. 420201) and prepared for intracellular staining using the True-Nuclear Transcription Factor Buffer Set (BioLegend, Cat. No. 424401).

#### 2.4.2 Intracellular staining

200 µl of cell True-Nuclear fixation buffer was added to each well for 45 minutes at room temperature and protected from light. Cells were then washed three times with 200 µl of True-Nuclear permeabilization buffer and samples resuspended in 100 µl of permeabilization buffer and stained with anti-human T-bet for 30 minutes are room temperature. Cells were washed three times with 200 µl of permeabilization buffer and resuspended in 150 µl of cell staining buffer.

#### 2.4.3 Data acquisition

Spectral reference controls (single color stains) and fluorescence minus one (FMO) controls were also prepared. Flow cytometry was performed using a 3-laser Cytek Aurora Spectral Cytometer. 50,000 events (live B cells) were acquired for each sample and data was analyzed using FCS Express 7.

### 2.5 Positive selection of CD11c^+^ B cells

Total B cells were isolated from frozen PBMC samples as stated above and prepared for positive selection of CD11c^+^ B cells using the MojoSort human anti- PE nanobeads (BioLegend Cat. No. 480091). Isolated B cells were washed in MojoSort buffer, blocked with Human TruStain FcX, and stained with anti-human CD11c-PE antibody and isolated according to the manufacturer’s instructions. Collected CD11c^-^ and CD11c^+^ B cells were then washed twice (350 x g for 10 minutes) with PBS. Purity was assessed by flow cytometry (**Supplementary Figure 1C**).

### 2.6 *In vitro* B cell culture

CD11c^+^ and CD11c^-^ B cells were isolated using magnetic bead separation (16). 2x10^3^ cells were plated with wells seeded with 3 x10^3^ MS-40L^lo^ cells (kindly provided by the Kelsoe Laboratory) per well and cultured in complete RPMI media in the presence of recombinant human cytokines IL-2 (50 ng/mL), IL-4 (10 ng/mL), IL-21(10 ng/mL) and BAFF (10 ng/mL) (all purchased from Peprotech) for 14 days (n = 6 per group). LPS was added to cytokine stimulated wells 48 hours prior to cell collection (n = 3). Cells were collected and stained with anti-CD20, anti-CD38, and anti-CD27 antibodies for analysis by flow cytometry. Culture supernatants were collected for analysis of IgG production by ELISA.

### 2.7 Human IgG ELISA

High bind 96 well plates were coated overnight with 2 ug/mL of anti-human IgG (Mabtech, clone MT91/145) diluted in PBS. Plates were blocked with PBS + 1% BSA for 1 hour at room temperature. Culture supernatants were inculcated at a dilution of 1:1000 for 2 hours at room temperature. IgG was detected using biotin-conjugated anti-human IgG (Mabtech, clone MT78/145) at a dilution of 1:128,000 in blocking buffer and incubated for 1 hour, followed by incubation with high sensitivity streptavidin-HRP (1:40,000) for 1 hour. The reaction was visualized by TMB substrate solution (Fisher Scientific) stopped with 1M H_2_SO_4_ and absorbance read at 450 nm. Sample concentrations were calculated based on optical density values of a standard curve of purified human IgG. Anti-AChR and anti-MuSK antibodies in day 14 culture supernatants from CD11c^+^ B cells stimulated with IL-2, IL-4, IL-21 and BAFF were detected using radioimmunoassay.

### 2.8 Statistical analysis

Data were analyzed by GraphPad Prism version 10.3.0. Data was tested for normality using Shapiro-Wilks test. Normally distributed data was compared using one-way ANOVA followed the Turkey’s test for multiple comparisons and data with non-normal distribution was compared non-parametric Kruskal-Wallis test followed by Dun’s *post-hoc* test for multiple comparisons. Spearman’s rank order correlation (rho) was used for correlation analyses. P values less than 0.05 were considered significant and shown as exact p-values. All error bars show standard error of the mean (^+^/_-_ SEM).

## 3 Results

### 3.1 DN and CD11c^+^ B cells are elevated in MG and comprise of several subsets

**Figure 1:**
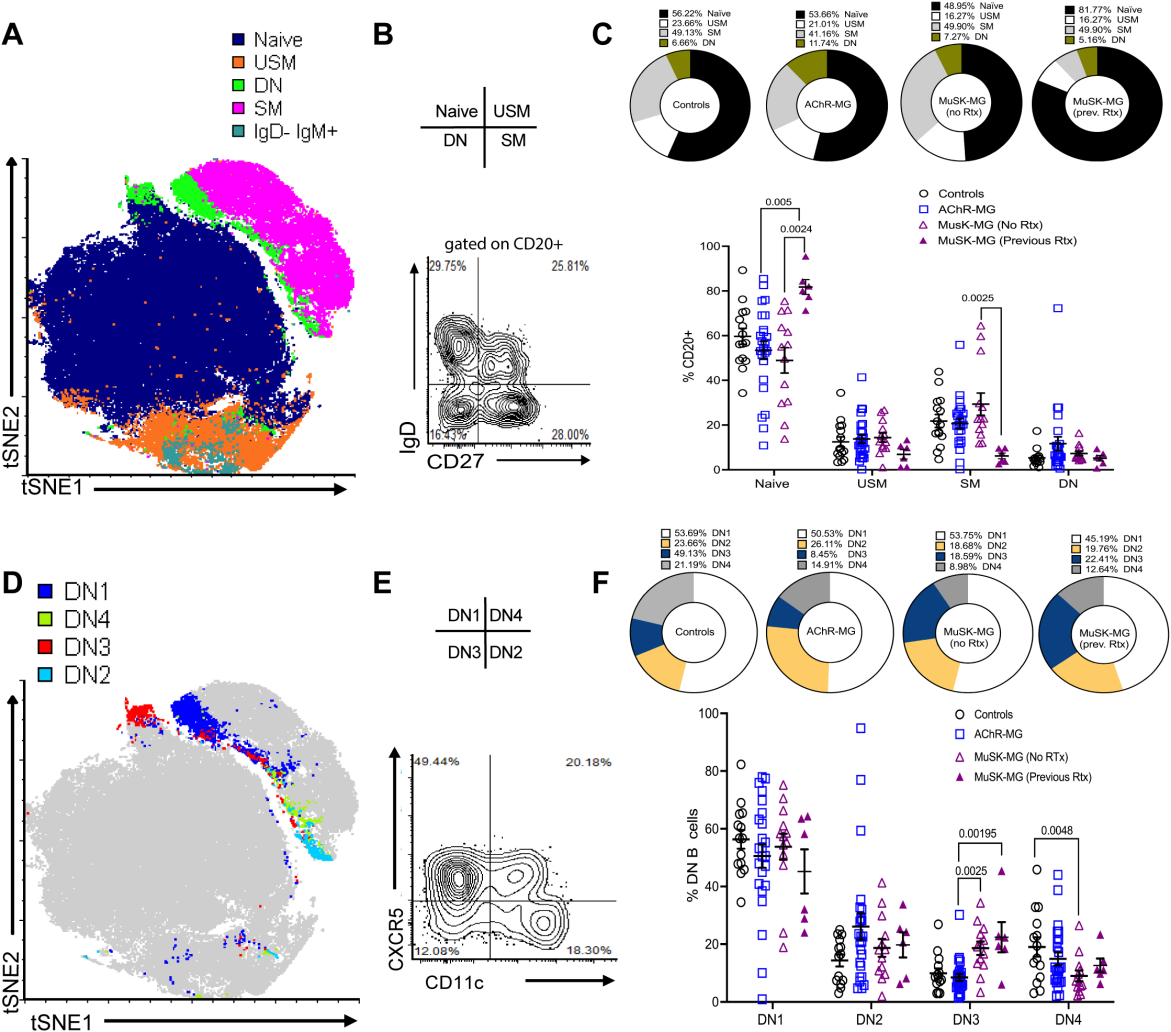
Distinct subsets of DN B cells are increased in AChR-MG and MuSK-MG. High-dimensional flow cytometry analysis of B cell populations. **(A)** Merged tSNE analysis of flow cytometry data from 3 controls, 6 AChR-MG, and 6 MuSK-MG subjects. **(B)** Flow cytometry plot showing IgD and CD27 expression on CD20^+^-gated B cells. **(C)** Average proportions of naïve (IgD^+^ CD27^−^), unswitched memory (USM, IgD^+^ CD27^+^), switched memory (SM, IgD^−^ CD27^+^), and double-negative (DN, IgD^−^ CD27^−^) B cells in each subject group (upper panel). Quantification of these subsets in controls (n = 14), AChR-MG (n = 24), and MuSK-MG subjects stratified by rituximab treatment status (rituximab-naïve n = 13, and rituximab-treated n = 6). (lower panel). **(D)** Merged tSNE analysis of DN B cell populations. **(E)** Representative flow cytometry plot showing DN B cell subset gating: DN1 (CXCR5^+^ CD11c^−^), DN2 (CXCR5^−^ CD11c^+^), DN3 (CXCR5^−^ CD11c^−^), and DN4 (CXCR5^+^ CD11c^+^). **(F)** Average proportions of DN B cell subsets for each subject group (upper panel) and quantification in controls, AChR-MG, and MuSK-MG subjects stratified by rituximab treatment status (lower panel). Data are represented as mean ± SEM. Normality was tested using the Shapiro-Wilk test. Exact p values ≤ 0.05 are displayed.

To identify the population of B cell subsets, we performed high-dimensional spectral flow cytometry to analyze the expression of 14 makers on B cells from AChR-MG (n = 24), MuSK-MG (n = 19), and non-autoimmune control subjects (n = 14). Six MuSK-MG subjects had undergone anti-CD20 therapy 18–36 months prior to analysis and were evaluated as a separate group. Subject characteristics are reported in **Table 1**. In a subset of patients (3 controls, 6 AChR-MG and 6 MuSK-MG subjects) we performed t-stochastic neighbor embedding (tSNE) analysis on CD3^-^ CD19^+^ CD20^+^ B cells to reduce dimensionality (**Figure 1A**) We identified naïve, unswitched memory, switched memory, IgD^-^ IgM^+^ memory, and double negative (DN) populations, with naïve B cells consisting of the largest population. Quantification of B cell subsets based on IgD and CD27 expression (**Figure 1B**) revealed no significant differences in the frequencies of naïve, unswitched memory, switched memory, or DN B cells between AChR-MG, rituximab-naïve MuSK-MG, and control subjects (**Figure 1C**). In contrast, previously rituximab-treated MuSK-MG subjects exhibited a significant increase in naïve B cells compared to both rituximab-naïve MuSK-MG and AChR-MG subjects. Additionally, previously treated MuSK-MG subjects showed a reduction in switched memory B cells compared to their rituximab-naïve counterparts.

Studies have revealed heterogeneity within the DN B cell population, identified based on differential expression of CD11c and CXCR5 (8, 13). To confirm these populations in our cohorts, DN B cells were categorized into DN1 (CXCR5^+^ CD11c^-^), DN2 (CXCR5^-^ CD11c^+^), DN3 (CXCR5^-^ CD11c^-^), and DN4 (CXCR5^+^ CD11c^+^) subsets (**Figures 1D, E**). DN2 and DN3 B cells have been reported to expand in autoimmune conditions and severe infection (8, 13, 15, 22). DN1 and DN2 B cells showed no significant differences between MG subtypes and controls. However, DN3 B cells were significantly increased in MuSK-MG compared to AChR-MG, underscoring their potential role in disease pathogenesis (**Figure 1F**). DN3 B cells remained significantly elevated in both rituximab-treated and untreated MuSK-MG subjects compared to AChR-MG. This suggests that DN3 B cells may not be effectively targeted to anti-CD20 treatment, or the DN3 B cell population can be effectively replenished by the naïve B cells.

**Figure 2:**
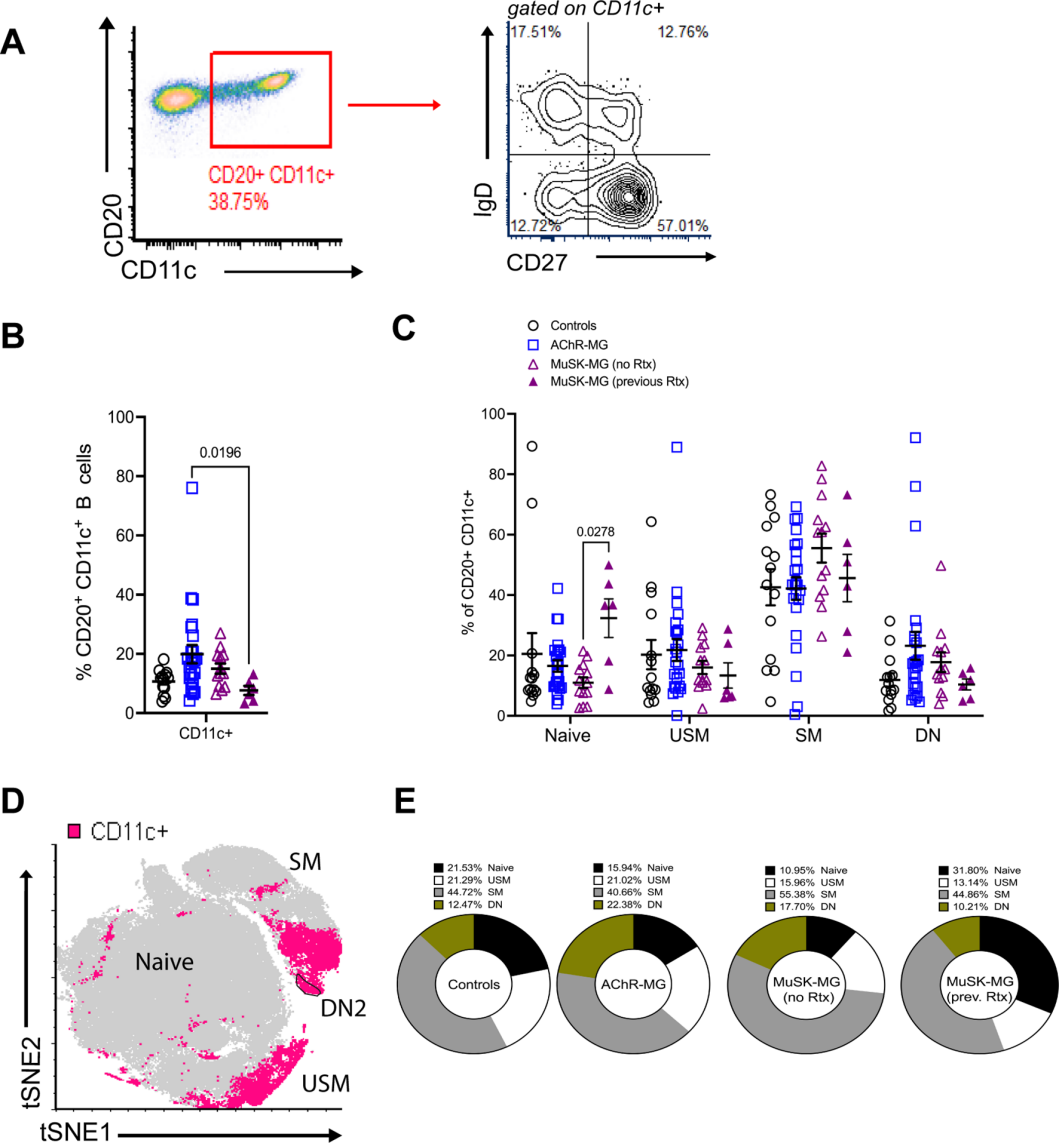
CD11c^+^ B cells are increased in AChR-MG and composed of memory B cells. Frequency of CD11c^+^ B cells in peripheral blood. **(A)** Flow cytometry plot displaying gating of CD20^+^ CD11c^+^ B cells followed by gating for IgD and CD27 expression. **(B)** Frequencies of CD20^+^ CD11c^+^ B cells for controls (n = 14), AChR-MG (n = 24), and MuSK-MG subjects stratified by rituximab treatment status (rituximab-naïve n = 13, and rituximab-treated n = 6). **(C)** Frequencies of CD11c^+^ B cells that are naïve (IgD^+^ CD27^−^), unswitched memory (USM, IgD^+^ CD27^+^), switched memory (SM, IgD^−^ CD27^+^), and double-negative (DN, IgD^−^ CD27^−^). **(D)** Merged tSNE analysis of DN B cell populations from 3 controls, 6 AChR-MG, and 6 MuSK-MG subjects, with CD11c^+^ B cells represented in pink. **(E)** Average proportions of naïve, unswitched memory, switched memory, and double-negative B cells in the CD11c^+^ B cell population. Data are represented as mean ± SEM. Normality was tested using the Shapiro-Wilk test. Groups were compared using the Kruskal-Wallis test with Dunn’s multiple comparison test. Exact p values ≤ 0.05 are displayed.

To address the B cell population for the frequency of other subsets of atBCs, we next examined the distribution of CD11c^+^CD20^+^ B cells (**Figure 2A**). We noted an elevation of CD11c^+^ B cells in AChR-MG subjects compared to rituximab-naïve MuSK-MG subjects (**Figure 2B**). To further characterize this population, we assessed the expression of IgD and CD27 to identify the populations within the CD11c^+^ compartment (**Figure 2C**) (23). The majority of CD11c^+^ B cells were comprised of switched memory B cells (23), with DN B cells making up only a small fraction (between 12.47% in controls, 17.70% in rituximab-naïve MuSK-MG, 10.21% in rituximab treated MuSK-MG, and highest in 22.38% in AChR-MG) (**Figures 2C–E**).

Together, our results show that CD11c^+^ B cells are heterogenous and consist mainly of memory B cells. The alterations observed in these subsets due to previous rituximab treatment suggest that while rituximab increases naïve B cells and reduces switched memory and CD11c^+^ B cells, its effects on DN3 B cell subsets are limited.

### 3.2 CD11c^+^ B cells and DN B cells associate with clinical severity and disease onset in MG

**Figure 3:**
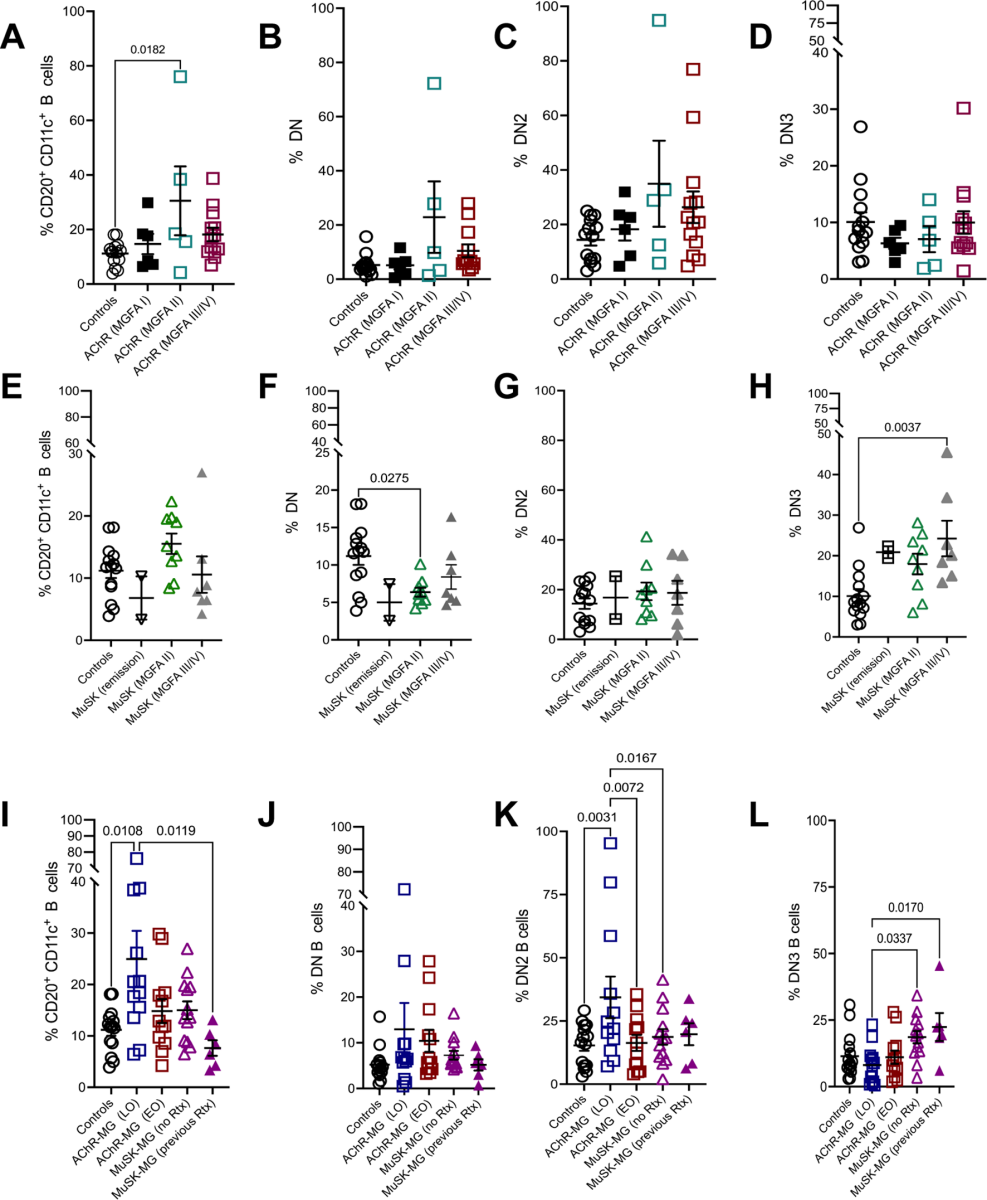
CD11c^+^ and DN B cell subsets associate with clinical severity and disease onset in MG. Frequency of CD20^+^ CD11c^+^ B cells **(A)**, DN B cells **(B)**, DN2 B cells **(C)**, and DN3 B cells **(D)** in AChR-MG subjects stratified based on MGFA classification. Frequency of CD20^+^ CD11c^+^ B cells **(E)**, DN B cells **(F)**, DN2 B cells **(G)**, and DN3 B cells **(H)** in MuSK-MG subjects stratified based on MGFA classification. Frequencies of CD20^+^ CD11c^+^ B cells **(I)**, DN B cells **(J)**, DN2 B cells **(K)**, and DN3 B cells **(L)** in non-autoimmune controls and AChR-MG subjects stratified by disease onset (EOMG = early-onset MG; LOMG = late-onset MG) and MuSK-MG stratified by rituximab treatment status. Data were tested for normality using the Shapiro-Wilk test. Normally distributed data were compared using one-way ANOVA with Tukey’s multiple comparison test. Non-normally distributed data were compared using the Kruskal-Wallis test with Dunn’s multiple comparison test. Exact p values ≤ 0.05 are displayed.

Patients with MG demonstrate diversity in clinical presentation, severity, and onset of disease. We stratified the AChR-MG subjects based on MGFA classification and assess their expression of atBC subsets. With disease severity, AChR-MG CD11c^+^ were elevated in moderate disease (MGFA Class II) but did not differ significantly between ocular (Class I) and severe (Class III/IV) cases (**Figures 3A, B**). DN2 and DN3 B cells showed no association with MGFA classification in AChR-MG (**Figures 3C, D**).

We then assessed the disease severity in MuSK-MG in association to atBC frequency. Of the six subjects previously treated with rituximab, two were in remission, while the remaining four subjects were symptomatic: MGFA Class III (n = 3) and MGFA Class II (n = 1). CD11c^+^ B cells followed a similar trend as AChR-MG, increasing in moderate disease (MGFA Class II), while DN B cells were significantly reduced compared to controls (**Figures 3E, F**). DN2 B cell frequencies remained stable across disease severity and were comparable to controls (**Figure 3G**). Interestingly, DN3 B cells were elevated across all MuSK-MG groups, including subjects in remission. The highest DN3 B cell frequencies were observed in severe disease, with significantly increased levels compared to controls (**Figure 3H**). Elevated levels of DN3 B cells suggest a potential role in disease progression or reflect underlying immune dysregulation in MuSK-MG.

To investigate whether atBC subsets are associated with disease variations, we stratified AChR-MG subjects based on MGFA classification and disease onset (late-onset [LOMG] and early-onset [EOMG]). In AChR-MG, LOMG subjects exhibited significantly higher levels of CD11c^+^ B cells compared to controls and rituximab-treated MuSK-MG subjects. DN2 B cells in LOMG were also significantly increased compared to controls, EOMG, and rituximab naive MuSK-MG. Total DN and DN3 B cell levels did not differ between LOMG and EOMG (**Figures 3I–L**). Together, our results show specific associations of atBC subsets with disease activity with increased population of CD11c^+^ B cells in AChR-MG and the DN3 in MuSK-MG. The DN2 B cell population increase in LOMG parallels the increase in the CD11c^+^ population, highlighting the contribution of DN2 in the overall CD11c^+^ population.

### 3.3 DN B cell subsets correlate with CD11c^+^ B cells

**Figure 4:**
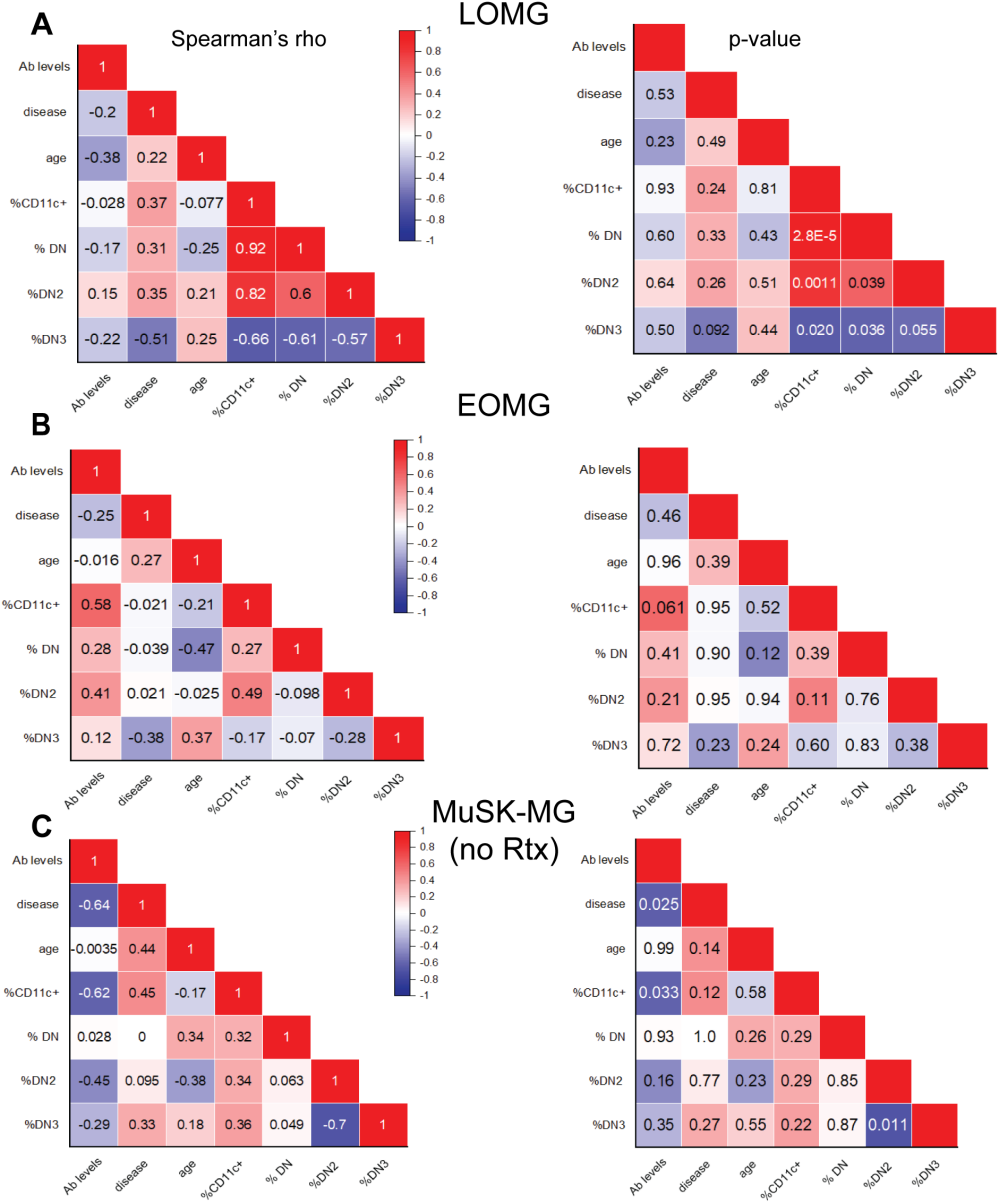
CD11c^+^ B cells correlate with DN B cell subsets. Correlation matrix of atypical B cell subset frequencies and clinical characteristics, including age, antibody levels, and disease duration. Left panels display Spearman’s rho values, with corresponding p-values shown on the right for **(A)** LOMG subjects, **(B)** EOMG subjects, and **(C)** rituximab-naïve MuSK-MG subjects. A two-tailed Spearman’s rank-order correlation test was performed. p-values < 0.05 are considered significant.

To assess the relationship between CD11c^+^ B cells and DN B cell subsets with clinical measures, we performed correlation analyses between atBC subset frequencies and age, autoantibody levels and disease duration. We observed no correlation between the atBCs and clinical measures in LOMG and EOMG (**Figures 4A, B**). While the rituximab-naïve MuSK-MG showed no correlation of atBCs with age and disease duration, the CD11c^+^ B cell subset demonstrated moderate negative association with autoantibody levels (r = -0.62, p=0.033). Whereas the rituximab treated MuSK-MG showed a negative correlation with age (r= -0.89, p=0.019) (**Supplementary Figure 2A**). The AChR-MG subjects evaluated in aggregate showed DN2 B cells with a moderate positive correlation with age (r = 0.41, p = 0.05) while the correlation was not present in control subjects (**Supplementary Figures 2B, C**).

In LOMG, CD11c^+^ B cells showed a strong and significant positive correlation with total DN (r = 0.92, p = 2.8 × 10^−5^) and DN2 B cells (r = 0.82, p = 0.0011) supporting the significance of DN2 to the CD11c^+^ population (**Figure 4A**). While similar trends were not observed between CD11c^+^ and DN B cells in EOMG and rituximab-naïve MuSK-MG subjects (**Figures 4B, C**). CD11c^+^ B cells and DN2 B cells in previously treated subjects have a strong correlation (r = 0.71), but did not reach significance (p = 0.11) due to the small number of samples in this group (**Supplementary Figure 2A**).

We then assessed the correlation of DN3 population in LOMG and found a negative correlation with CD11c^+^ (r = -0.66, p = 0.020), DN (r = -0.61, p = 0.036) and DN2 (r = -0.57, p = 0.055) (**Figure 4A**). DN3 and DN2 B cells exhibited a strong inverse correlation in rituximab-naive MuSK-MG (r = -0.70, p = 0.011) (**Figure 4C**). MuSK-MG subjects with prior rituximab treatment showed a moderate but insignificant negative correlation (r= -0.54, p=0.27) (**Supplementary Figure 2A**), suggesting that DN3 expansion is intrinsic to MuSK-MG pathology rather than a treatment-related effect. These findings highlight distinct relationships between DN B cell subsets that lack CXCR5 homing mechanism and support distinct immunological mechanisms that determine the expression of CD11c.

### 3.4 CD20 and FCRL5 expression in atypical B cells differentiates MG subtypes

**Figure 5:**
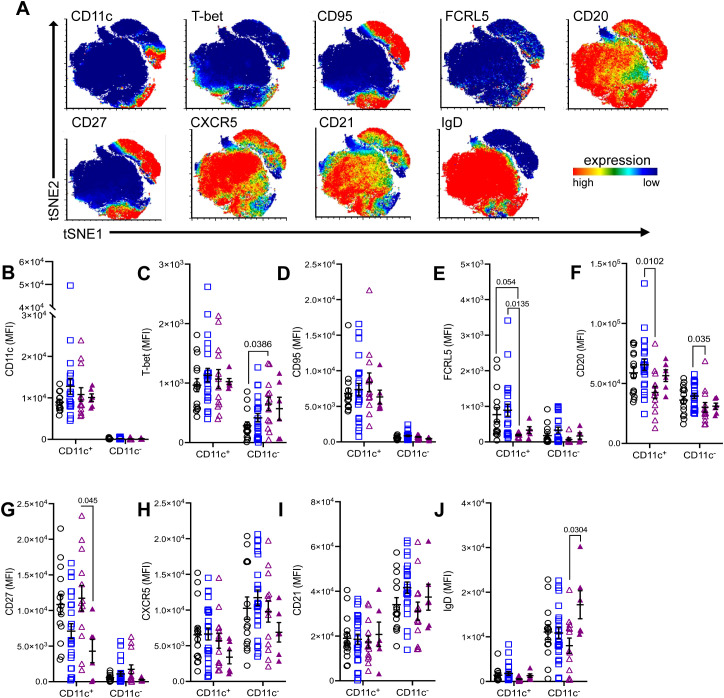
Differential expression of atypical B cell markers in CD11c^+^ B cells between MG subtypes. **(A)** Merged tSNE plots showing expression of select markers. Comparison of mean fluorescence intensity (MFI) of **(B)** CD11c, **(C)** T-bet, **(D)** CD95, **(E)** FCRL5, **(F)** CD20, **(G)** CD27, **(H)** CXCR5, **(I)** CD21, and **(J)** IgD between CD11c^+^ and CD11c^−^ B cells in non-autoimmune controls, AChR-MG, and MuSK-MG subjects stratified by rituximab treatment status. Data are represented as mean ± SEM. Normality was tested using the Shapiro-Wilk test. Mean expression was compared using the Kruskal-Wallis test with Dunn’s multiple comparison test. Exact p values ≤ 0.05 are displayed.

To identify differences in CD11c^+^ B cell phenotype between controls, AChR-MG, and MuSK-MG, we assessed the expression of markers that define atypical B cells using flow cytometry (**Figure 5A**). When compared to CD11c^-^ B cells, the CD11c+ B cells expressed patterns consistent with atBCs, which includes increased expression of CD11c, T-bet, CD95, FCRL5, CD20, and CD27 and reduced expression of CXCR5, CD21, and IgD (**Figures 5B–J**). Expression levels across all markers showed some variation, which may reflect the heterogeneity in the CD11c^+^ population. Overall increased expression of CD27 (**Figure 5G**) confirms CD11c^+^ B cells are mainly memory B cells, supported by the predominance of switched memory B cells (**Figure 2C**). In contrast, rituximab treated MuSK-MG subjects showed significantly reduced CD27 expression in CD11c^+^ B cells when compared to untreated MuSK-MG subjects (**Figure 5G**), likely reflecting the increased frequency of naïve cells in the CD11c^+^ B cell population (**Figure 2C**).

When comparing the three subject groups, CD11c^+^ B and CD11c^-^ B cells did not differ significantly between MG and controls; however, there were notable differences between MG subtypes. Interestingly, while T-bet expression is greater in CD11c^+^ B cells, we observed T-bet expression was significantly higher in CD11c^-^ B cells MuSK-MG subjects compared to controls (**Figure 5C**). Further analysis of CD11c^-^ T-bet^+^ cells determined these cells are CXCR5^+^, IgD^+^, and lack CD95 expression, indicating they are naïve B cells (data not shown). The increased IgD expression in CD11c^-^ B cells (**Figure 5J**) also points towards an expanded pool of naïve B cells following treatment, while reduced CXCR5 expression (**Figure 5H**) suggests that CD11c^−^ B cells may be redirected toward an extrafollicular response or a DN3 B cell phenotype.

Additionally, AChR-MG subjects showed higher expression FCRL5 in CD11c^+^ B cells compared to controls and rituximab-naive MuSK-MG patients, demonstrating that these cells may have higher activation rate as overexpression interferes with anergy of the cell (24)(**Figure 5E**). Notably, AChR-MG subjects exhibited significantly higher CD20 expression in both CD11c^+^ and CD11c^−^ B cells compared to MuSK-MG subjects (**Figure 5F**). These findings suggest that differential expressions of CD20 and FCRL5 in atBCs are distinguishing features between AChR-MG and MuSK-MG subtypes.

### 3.5 MG subtypes can be distinguished by CD20 expression in atypical B cells

**Figure 6:**
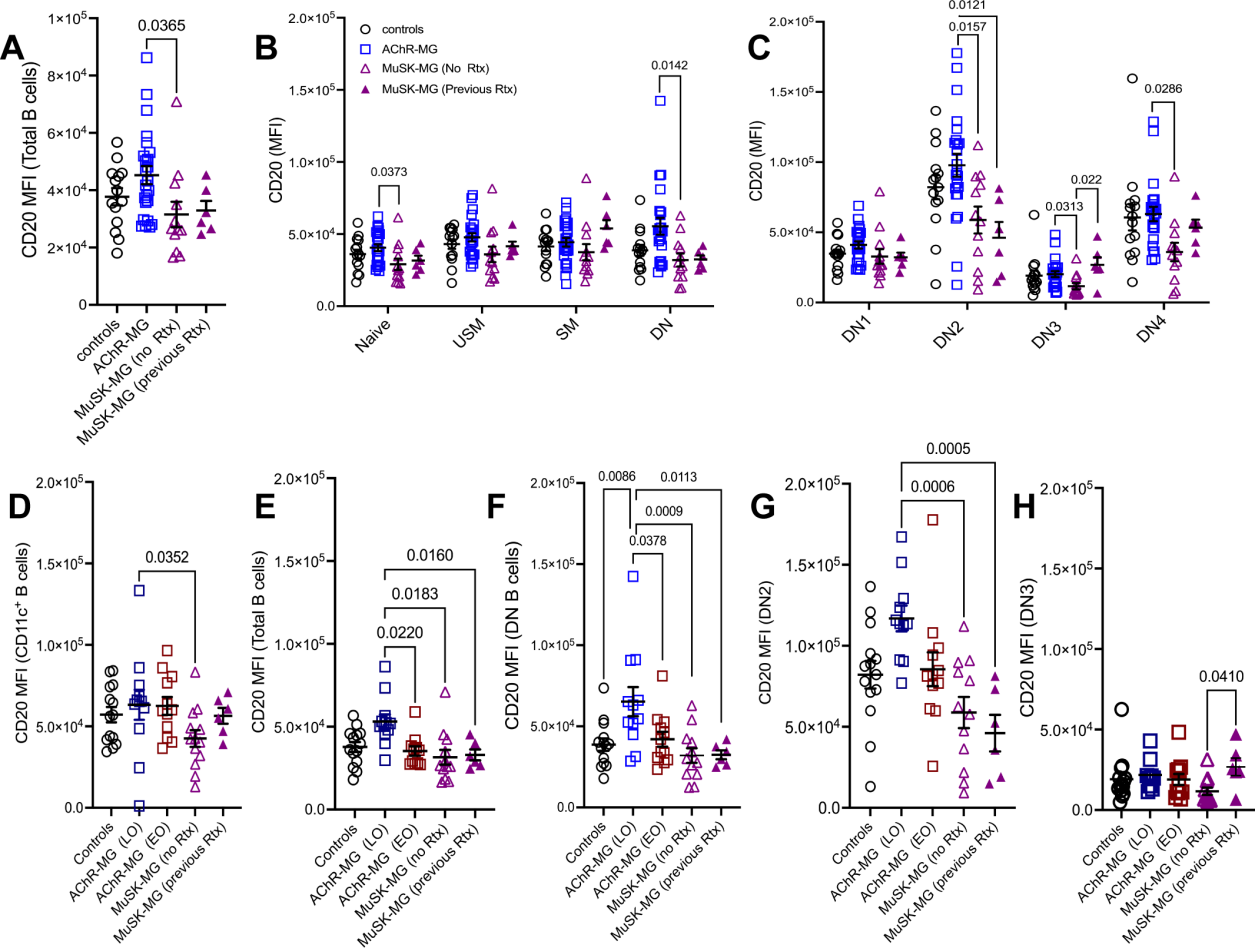
CD20 expression in atypical B cells distinguishes AChR-MG and MuSK-MG. Mean fluorescence intensity (MFI) of CD20 expression across B cell populations. CD20 MFI in **(A)** total CD20^+^ B cells; **(B)** naïve, unswitched memory, switched memory, and DN B cells; and **(C)** DN1, DN2, DN3, and DN4 B cell subsets in non-autoimmune controls, AChR-MG, and MuSK-MG subjects stratified by rituximab treatment status (rituximab-naïve and rituximab-treated). CD20 MFI in **(D)** CD11c^+^ B cells; **(E)** total CD20^+^ B cells; **(F)** DN B cells; **(G)** DN2 B cells; and **(H)** DN3 B cells in non-autoimmune controls, AChR-MG subjects stratified by disease onset (EOMG = early-onset MG; LOMG = late-onset MG), and MuSK-MG. Data are represented as mean ± SEM. Normality was tested using the Shapiro-Wilk test. Normally distributed data were compared using one-way ANOVA with Tukey’s multiple comparison test. Non-normally distributed data were compared using the Kruskal-Wallis test with Dunn’s multiple comparison test. Exact p values ≤ 0.05 are displayed.

To assess whether CD20 downregulation occurred across all B cell populations, we compared CD20 expression in total CD20^+^ B cells and several populations, including naïve, unswitched memory, switched memory, and DN B cells. Compared to AChR-MG, CD20 expression was reduced in total CD20^+^ B cells, naïve B cells, total DN B cells, DN2, DN3, and DN4 B cells in rituximab-naïve MuSK-MG subjects, whereas CD20 expression was not significantly different between rituximab-treated and AChR-MG subjects. Unswitched and switched memory B cells showed no reduction in CD20 expression in either MuSK-MG group (**Figures 6A–C**). In contrast, CD20 expression in DN2 B cells was unaffected by rituximab and remained significantly lower in both MuSK-MG groups compared to AChR-MG or controls (**Figure 6C**). CD20 expression did not differ significantly between rituximab-treated and treatment-naïve MuSK-MG subjects across most B cell populations, except for DN3 B cells, where CD20 expression was significantly higher in previously treated patients.

We also observed that CD11c-expressing populations (DN2, DN4) showed overall highest expression of CD20 when compared to all other B cell subsets. In contrast, DN3 B cells displayed the lowest (**Figure 6C**). The differential expression of CD20 among B cell subsets suggests that these subsets may be differentially targeted by anti-CD20 treatment.

We next examined whether elevated CD20 expression in AChR-MG was present in both LOMG and EOMG. Interestingly, CD20 expression in CD11c^+^ B cells did not differ between LOMG and EOMG (**Figure 6D**). However, significantly higher CD20 expression was observed in total B cells and DN B cells from LOMG subjects compared to both EOMG subjects and MuSK-MG subjects, independent of previous rituximab treatment (**Figures 6E, F**). No significant differences in CD20 expression in DN2 B cells were observed between LOMG and EOMG; however, LOMG subjects exhibited significantly higher CD20 expression compared to MuSK-MG subjects, regardless of rituximab treatment status (**Figure 6G**).

Together, our findings demonstrate that LOMG, EOMG, and MuSK-MG can be distinguished by CD20 expression in atBC subsets. Moreover, we show that B cell depletion may restore CD20 expression in certain B cell subsets, particularly CD11c^+^ B cells B cells and DN3 (**Figure 6H**), while sparing DN2 B cells. This differential effect on CD20 expression could have implications for the efficacy of subsequent anti-CD20 treatments.

### 3.6 Distinct co-expression signatures of CD11c^+^ B cells reveal MG subtype-specific immune responses

**Figure 7:**
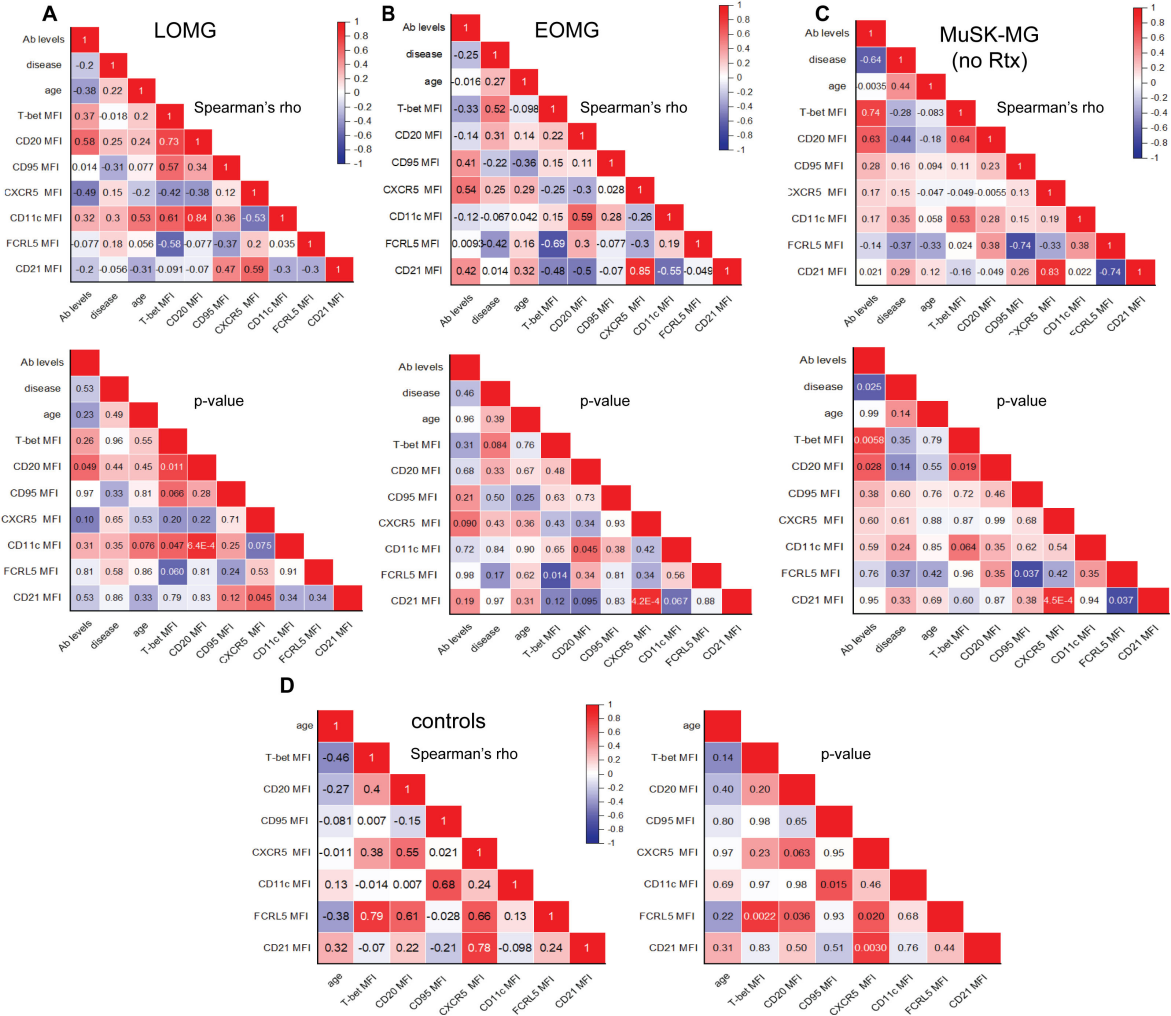
Distinct Co-Expression Signatures of atypical B cell markers in CD11c^+^ B Cells in MG Subtypes and Correlations with Clinical Characteristics. Correlation matrix of atypical B cell marker expression on CD11c^+^ B cells with antibody levels, disease duration, and age. Top panels display Spearman’s rho values, with corresponding p-values shown below for **(A)** LOMG, **(B)** EOMG, **(C)** rituximab-naïve MuSK-MG. In non-autoimmune control subjects, correlation matrix on the left displays Spearman’s rho values, with corresponding p values on the right **(D)**. A two-tailed Spearman’s rank-order correlation test was performed. P values <0.05 are considered significant.

We next assessed the relationships between expression levels of atypical markers in CD11c^+^ B cells and observed distinct patterns across MG subtypes. We observed a consistent positive correlation between CXCR5 and CD21 expression on CD11c^+^ B cells in LOMG (r = 0.59, p = 0.045), EOMG (r = 0.85, p = 4.2 x10^-4^), rituximab-naïve MuSK-MG (r = 0.83, p = 4.5 × 10^−4^), and controls (r= 0.78, p = 0.003, **Figure 7**) which is consistent with the downregulation of these markers (**Figures 5H, I**). Of interest is that the correlation did not persist with the rituximab treatment in MuSK-MG (**Supplementary Figure 2**). Similarity was also shown for correlation between CD11c and CD20 expression in LOMG (r=0.84, p= 6.4 x10^-4^) (**Figure 7A**) and EOMG (r = 0.59, p = 0.045) (**Figure 7B**). These findings support our observation that high CD20 expression is associated with CD11c-expressing populations.

T-bet expression in CD11c^+^ B cells showed a strong positive correlation with CD20 in LOMG (r = 0.73, p = 0.011) and treatment naïve MuSK-MG (r = 0.64, p = 0.019) (**Figures 7A, C**). In rituximab treated MuSK-MG subjects, T-bet and CD20 expression remained strongly correlated (r = 0.94, p = 0.048) (**Supplementary Figure 3**). In LOMG, T-bet also showed a positive correlation with CD11c expression (r = 0.61, p = 0.047), with a similar trend observed in rituximab-naïve MuSK-MG subjects (r = 0.53, p = 0.064).The positive correlations between CD20 and T-bet, CD20 and CD11c, and CXCR5 and CD21 across MG subtypes suggest that the co-expression of these markers in CD11c^+^ B cells may be driven by shared regulatory mechanisms.

Distinct co-expression patterns were also identified distinct from non-autoimmune controls. In MuSK-MG, FCRL5 expression unique associations with CD95 and CD21, a pattern not observed in LOMG or EOMG. We showed that in naïve MuSK-MG, FCRL5 inversely correlated with CD95 (r = -0.74, p = 0.037) and CD21 (r = -0.74, p = 0.037) (**Figure 7C**) which was maintained in the rituximab treated MuSK-MG (**Supplementary Figure 3**). In contrast, in EOMG subjects, FCRL5 expression was inversely correlated with T-bet expression (r = -0.69, p = 0.014) (**Figure 7B**). The FCRL5 expression pattern in non-autoimmune controls was positively correlated with T-bet (r = 0.79, p = 0.0022), CD20 (r =0.61, p = 0.036), and CXCR5 (r = 0.66, p = 0.02) (**Figure 7D**). Together, these findings suggest that the distinct co-expression patterns associated with the inhibitory receptor FCRL5 may reflect the ability of the CD11c^+^ B cells to respond to activating signals within their unique immune environments, shaping the dysregulation in MG subtypes.

We further examined the relationship between CD11c^+^ B cell marker expression and clinical characteristics, including antibody levels, disease duration, and age. In LOMG, CD20 expression in CD11c^+^ B cells positively correlated with AChR antibody levels (r = 0.58, p = 0.049) (**Figure 7A**), suggesting a potential link between CD11c^+^ B cells and sustained autoantibody production in this subtype. In EOMG, T-bet expression in CD11c^+^ B cells showed a positive trend with disease duration (r = 0.54, p = 0.064), suggesting that T-bet expression may gradually increase as disease progresses (**Figure 7B**).

Relationships were also observed between CD20 expression in DN2 B cells and clinical characteristics in AChR-MG. In LOMG, CD20 expression in DN2 (r= 0.7958, p = 0.0031) B cells strongly correlated with disease duration (**Supplementary Figure 4A**). In EOMG, DN2 B cells (r = 0.6325, p = 0.0368) positively associated with AChR antibody levels (**Supplementary Figure 4B**).

In rituximab-naïve MuSK-MG, we observed strong positive correlations between T-bet (r = 0.74, p = 0.0058) and CD20 (r = 0.63, p = 0.028) expression in CD11c^+^ B cells and antibody levels (**Figure 7C**). Conversely, in rituximab-treated MuSK-MG subjects, these relationships were reversed, with T-bet (r = -0.93, p = 0.0077) and CD20 (r = -0.81, p = 0.050) expression in CD11c^+^ B cells negatively correlating with antibody levels. These findings, together with the reduced frequency of CD11c^+^ B cells in rituximab-treated patients (**Figure 2B**), provide further evidence that CD11c^+^ B cells may act as precursor antibody-secreting cells that are targeted by anti-CD20 therapy.

### 3.7 CD11c^+^ B cells differentiate into CD27^++^ CD38^++^ antibody secreting cells and respond to LPS stimulation

Given the link between atBCs and antibody levels in MG, we next conducted functional assays to assess CD11c^+^ B cells differentiation into ASCs that secrete antibodies. Information on the subjects corresponding to the samples used in these experiments can be found in **Table 2**.

**Table 2:** Subject information for *in vitro* functional assays.

Stimulation	Sample	Onset	Sex	Age	Treatment	Disease duration
• CD40L • cytokines (IL-2, IL-4, IL-21, BAFF)	HC1		M	56		
	HC2		F	27		
	HC3		F	56		
	AChR1	EO	F	23	pyridostigmine	4 years
	AChR2	LO	M	71	none	2 years
	AChR3	LO	F	58	mestinon	9 years
	MuSK1		M	32	previous rtx	4 years
	MuSK2		F	21	mestinon	3 months
	MuSK3		F	62	previous rtx	44 years
• CD40L • cytokines (IL-2, IL-4, IL-21, BAFF) • LPS	HC4		F	23		
	HC5		F	52		
	HC6		F	39		
	AChR4	EO	F	28	pyridostigmine	3 years
	AChR5	EO	F	47	none	6 years
	AChR6	EO	F	33	mestinon	15 years
	MuSK4		F	23	previous rtx	8 years
	MuSK5		F	22	none	3 mon
	MuSK6		F	30	none	3 years

We stimulated CD11c^+^ B cells or CD11c^-^ B cells with CD40L and cytokines IL-2, IL-21, IL-4, and BAFF (25) to produce a T cell dependent B cell activation. Here, we assessed response to cytokine stimulation in six subjects per group. The AChR-MG group consisted of both LOMG (n = 2) and EOMG (n = 4) subjects. To demonstrate differences in CD11c^+^ B cells to respond to TLR signals, we tested LPS addition to the cultures at 48 hours prior to cell harvest in three of the six subjects, with the AChR-MG group consisting of only EOMG subjects. Cell viability for both CD11c^+^ and CD11c^−^ B cell cultures remained high, averaging approximately 75–85% at Day 7 and 60–70% by Day 14. CD11c expression was maintained at Day 7, with an average of 88% CD11c^+^ cells, but declined to an average of 55% by Day 14.

**Figure 8:**
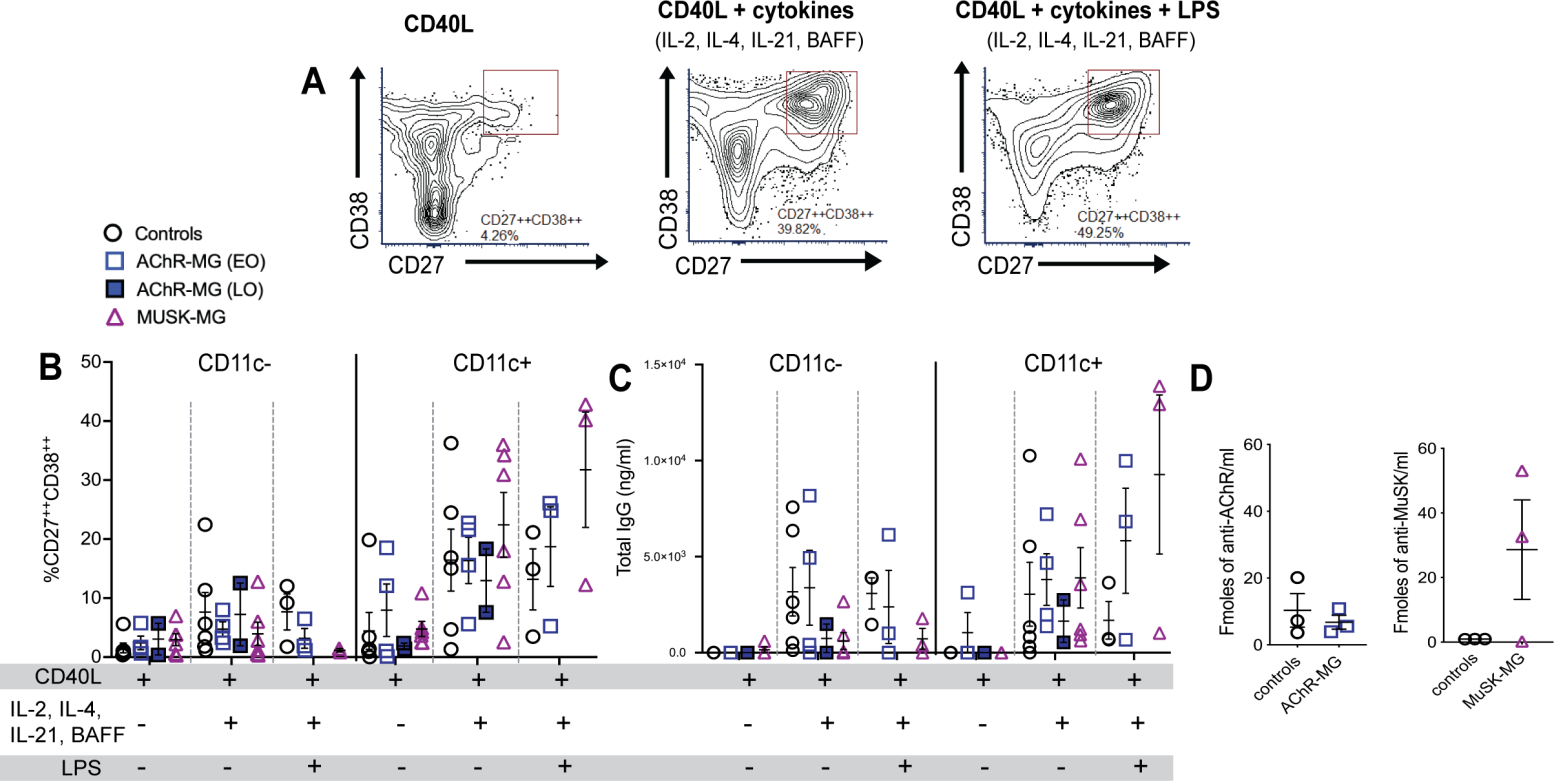
CD11c^+^ B cells differentiate into ASCs and produce autoantibodies in MuSK-MG. Isolated CD11c^−^ and CD11c^+^ B cells were stimulated with CD40L, IL-2, IL-12, and BAFF (n = 6 for each group) for 14 days. AChR-MG subjects were stratified based on disease onset (LOMG n = 2, EOMG n = 4). In LPS-treated wells, cells were stimulated with CD40L and cytokines (IL-2, IL-4, IL-21, and BAFF) for 14 days, with LPS added 48 hours prior to harvest (n = 3). **(A)** Representative flow cytometry plots of CD11c^+^ B cells from a MuSK-MG subject. Antibody-secreting cells (ASCs) were identified as CD27^++^CD38^++^ cells. **(B)** Quantification of CD11c^−^ and CD11c^+^ B cells that differentiated into ^+^CD27^++^CD38^++^ ASCs. **(C)** Total IgG production (ng/mL) in culture supernatants as measured by ELISA. **(D)** Radioimmunoassay measuring anti-AChR and anti-MuSK antibody levels in culture supernatants from CD11c^+^ B cells (fmol/mL) stimulated with CD40L, IL-2, IL-4, IL-21 and BAFF for 14 days. Data are represented as mean ± SEM.

After 14 days of culture, cells were analyzed by flow cytometry for CD27 and CD38 expression (**Figure 8A**) and supernatants analyzed by ELISA for IgG production. We observed greater frequencies of CD27^++^CD38^++^ ASCs in CD11c^+^ B cell cultured with cytokines IL-2, IL-4, IL-21, and BAFF both in the presence and absence of LPS, compared to CD11c^-^ B cells in all groups, confirming previous reports that CD11c^+^ B cells differentiate into ASCs (**Figures 8B, C**). CD11c^+^ B cells from MuSK-MG stimulated with cytokines and LPS had demonstrated a greater capacity to differentiate into ASCs (**Figure 8B**). Total levels of IgG in culture supernatants were also higher in CD11c^+^ B cell cultures compared to CD11c^-^ B cells (**Figure 8C**). We also observed a strong positive correlation between the frequency of CD27^++^CD38^++^ cells and total IgG production in CD11c^+^ B cell cultures from MuSK-MG subjects (*r* = 0.83, *p* = 0.0409), but not from AChR-MG subjects (*r* = 0.26, *p* = 0.6181).

In ASC differentiation and IgG production, CD11c^+^ B cells from both AChR-MG and MuSK-MG showed an enhanced response to LPS. The non-autoimmune controls demonstrated reduced differentiation and IgG secretion with LPS stimulation when compared to stimulation with CD40L and cytokines alone. Interestingly, the CD11c^+^ B cells from 2 out of 3 MuSK-MG subjects produced high levels of anti-MuSK antibodies in response to CD40L and cytokines, whereas CD11c^+^ B cells from AChR did not produce levels of AChR antibodies that were different from controls (**Figure 8D**). These findings suggest that CD11c^+^ B cells in MuSK-MG are either more primed for ASC differentiation or respond to distinct signaling requirements for autoantibody production compared to AChR-MG.

## 4 Discussion

Our study provides evidence that atypical B cell (atBC) heterogeneity in MG is shaped by disease-specific immune responses. We identified CD11c^+^ and DN2 B cells as enriched in AChR-MG, particularly in late-onset MG (LOMG). MuSK-MG B cells were significantly elevated in DN3 B cells population and correlated with disease severity. CD20 expression emerged as a distinguishing feature, with reduced expression across MuSK-MG B cell subsets. Functionally, CD11c^+^ B cells from MuSK-MG exhibited greater differentiation into antibody-secreting cells (ASCs), contributing to autoantibody production. Our findings shed new insights into the mechanisms driving MG pathogenesis and have broader implications for other IgG1–3 and IgG4-mediated autoimmune diseases.

The expansion of CD11c^+^ and DN2 B cells in IgG1–3 mediated AChR-MG, particularly in LOMG, may reflect age-related immune changes, as atBCs are known to increase with age (26). The moderate correlation between DN2 B cells and age in AChR-MG suggests that immune aging may drive their expansion in LOMG. In contrast, the reduced levels of CD11c^+^ and DN2 B cells in EOMG suggest that distinct mechanisms underlie B cell dysregulation, supporting previous studies suggesting that EOMG and LOMG arise from distinct immunological mechanisms (27–29). EOMG is characterized by thymic pathology, marked by B cell follicles that correlate with circulating AChR antibodies (30) and increased TLR and IFN expression (31, 32). Given the established role of IFN and TLR signaling in atBC activation, we speculate that this localized inflammation may drive atBC differentiation within the thymus, reducing their presence in circulation. The positive correlation between T-bet expression in CD11c^+^ B cells and disease duration in EOMG further supports their role in disease progression and IFN responsiveness. Future studies should address potential changes in atBC population after thymectomy in EOMG.

The enrichment of DN3 B cells in MuSK-MG and IgG4-RD (13), suggests a shared immune mechanism across IgG4-mediated conditions. Research on DN3 B cells is limited with no clear function of this population. DN3 B cells have been identified in severe COVID-19 and SLE, which may highlight their potential role in broader immune dysregulation (7, 15, 33). Findings in SLE, where low CD19 expression in DN3 B cells has been linked to short-lived plasmablasts, may also characterize MuSK-MG (7). Although we did not directly assess DN3 B cell-plasmablast relationships in MuSK-MG, our findings raise the possibility that DN3 B cells support IgG4 antibody production in MuSK-MG.

CD20 expression emerged as a key feature distinguishing MuSK-MG. We observed that CD20 across nearly all B cell subsets showed reduced expression, including DN3 B cells. This broad downregulation may impair the ability of anti-CD20 targeted therapies to effectively deplete pathogenic B cell subsets. The persistence of DN3 B cells in rituximab-treated MuSK-MG subjects supports this, suggesting that low CD20 expression may contribute to expansion of the population and relapse, although we cannot discount the repopulation from the naïve B cell compartment. Given the correlation between DN3 B cells and disease severity in MuSK-MG, alternative therapeutic strategies may be necessary to target this population.

In contrast, CD11c^+^ B cells were significantly reduced in rituximab-treated MuSK-MG patients, suggesting that these cells are effectively targeted by anti-CD20 treatment. Importantly, our data showed that adjusting for rituximab treatment showed a correlation between CD20 and T-bet expression in CD11c^+^ B cells and MuSK antibody levels, underscoring the role of CD11c^+^ B cells in sustaining pathogenic antibody responses.

Lastly, we sought to evaluate the functional heterogeneity of CD11c^+^ B cells. Our *in vitro* analysis confirms that atBCs are primed for differentiation into ASCs and require T cell-dependent signals such as CD40L, IL-2, IL-21, and IL-4. CD11c^+^ B cells from MuSK-MG exhibited the strongest differentiation into antibody-secreting cells in response to stimulation compared to AChR-MG and controls, suggesting distinct functional capacities between MG subtypes. The addition of LPS as a TLR4 agonist increased the differentiation into CD27^++^CD38^++^ ASCs in both the AChR-MG and MuSK-MG samples with a correlation to increase IgG production, whereas the LPS in controls reduced the effect. The heightened innate immune response supports the dysregulation of TLR4 in AChR-MG (28, 31) and offers clues to the activation in MuSK-MG. We found a reduced expression of FcRL5 on CD11c^+^ B cells from MuSK-MG subjects which may suggest that these cells are able to secrete more antibodies compared to FcRL5+ atypical B cells (34). Notably, we found that CD11c^+^ B cells from MuSK-MG produced MuSK antibodies, whereas AChR antibody levels did not differ between AChR-MG and controls.

While our study highlights intriguing correlations of atBCs subsets with MG subtypes, it does have limitations. While we extensively characterized CD11c^+^ B cells, we did not fully evaluate DN B cell functionality. Given the overlap between DN2 and CD11c^+^ B cells, this population may have functional redundancy in AChR-MG. Although we sought to better understand DN3 B cells in MuSK-MG, their correlation with disease severity did not extend to disease duration or antibody levels. DN3 B cells, in general, remain poorly characterized, and their exact defining features are still unclear, which limits our ability to understand their role in an autoimmune disease. Additionally, our small sample size for rituximab-treated MuSK-MG patients limits our ability to draw firm conclusions about the long-term effects of treatment on atBC populations. Future studies with paired pre- and post-treatment samples will be essential to addressing this question.

Our findings highlight that the implications of atBC heterogeneity in autoimmune diseases are only beginning to emerge, with several important questions remaining unanswered. One key question concerns the relationship between atBC subsets. The presence of multiple atBC subsets, each linked to distinct disease parameters, suggests that no single subtype drives disease pathology. For example, while DN3 B cells in MuSK-MG correlated with disease severity, they were not associated with antibody levels or disease duration. Conversely, although CD11c^+^ B cells were not significantly elevated compared to controls or AChR-MG, they demonstrated functional significance by contributing to autoantibody production in MuSK-MG.

In summary, our study demonstrates that atBCs are a heterogeneous population with distinct phenotypic and functional profiles across MG subtypes. CD20 expression emerged as a key feature of atBCs, correlating with autoantibody levels and disease duration, with differential expression patterns distinguishing AChR-MG and MuSK-MG. Notably, the variation in CD20 expression among DN B cell subsets, coupled with the observed impact of rituximab on atBC populations, offers new insights into the therapeutic potential of targeting atBCs. The reduced CD20 expression in MuSK-MG, particularly in DN3 B cells, may limit the effectiveness of anti-CD20 therapies and contribute to persistent immune dysregulation. Conversely, CD11c^+^ B cells in AChR-MG, which are enriched in patients with moderate disease, may be more effectively targeted in early-stage treatment strategies. These insights highlight the importance of tailored therapeutic approaches that account for atBC heterogeneity and provide a foundation for future investigations into the role of atBCs in other autoimmune diseases.

## Data Availability

The original contributions presented in the study are included in the article/**Supplementary Material**. Further inquiries can be directed to the corresponding author.
