# Supplementary material for: Subtype-specific atypical B cell profiles in myasthenia gravis reveal distinct immunopathological pathways
Source: Front Immunol. 2025 Jun 18;16:1608160. doi: 10.3389/fimmu.2025.1608160 (PMC12213399; doi:10.3389/fimmu.2025.1608160)
Supplement: Supplementary file 1 [file DataSheet1.docx]

**Supplementary Figures**

**Supplementary Figure 1. Purity of isolated B cells and CD11c⁺ B cells.**
(A) Gating strategy used for B cell isolation. (B) Assessment of total B cell purity following negative selection. (C) Assessment of CD11c⁺ B cell purity following positive selection.

**Supplementary Figure 2. Relationships between atypical B cell frequencies, age, antibody levels, and disease duration in AChR-MG.** Correlation matrix of frequencies of atypical B cell subsets and clinical characteristics of age, antibody levels, and disease duration. Left panels show Spearman’s rho values and corresponding p values on the right for (A) MuSK-MG subjects previously treated with rituximab (B) AChR-MG subjects in aggregate, and (C) non-autoimmune controls. Two tailed Spearman’s rank-order correlation test was performed. P values < 0.05 are considered significant.

**Supplementary Figure 3. Correlation analysis in MuSK-MG subjects previously treated with rituximab.** Correlation matrix of atypical B cell subset frequencies and clinical characteristics, including age, antibody levels, and disease duration. (A) Spearman’s rho values, with (B) corresponding p-values shown for MuSK-MG subjects previously treated with rituximab (n = 6). Correlation matrix of CD20 expression in atypical B cell subsets (CD11c⁺ B cells, DN B cells, DN2 B cells, and DN3 B cells) and clinical characteristics, including antibody levels, disease duration, and age. (C) Spearman’s rho values and (D) corresponding p-values for MuSK-MG subjects previously treated with rituximab. P values < 0.05 are considered significant.

**Supplementary Figure 4. CD20 expression in** **DN B cell subsets correlate with disease duration and antibody levels in LOMG and EOMG.** Correlation of CD20 expression in DN2 B cells and disease duration (A) and AChR antibody levels (B) in LOMG and EOMG subjects. A two-tailed Spearman’s rank-order correlation test was performed. P values < 0.05 are considered significant.
