# Supplementary material for: Subtype-specific atypical B cell profiles in myasthenia gravis reveal distinct immunopathological pathways
Source: Front Immunol. 2025 Jun 18;16:1608160. doi: 10.3389/fimmu.2025.1608160 (PMC12213399; doi:10.3389/fimmu.2025.1608160)
Supplement: Supplementary file 2 [file Table1.docx]

| Materials | | |
| --- | --- | --- |
| FLOW CYTOMETRY | | |
| **Antibody** | **Clone** | **Manufacturer** |
| anti-human CD3-BV650 | UTHT1 | BioLegend |
| anti-human CD20-Alexa Fluor 700 | 2H7 | BioLegend |
| anti-human CD27-BV605 | O323 | BioLegend |
| anti-human CD21-PE-Cy7 | Bu32 | BioLegend |
| anti-human CD86-BV785 | IT2.2 | BioLegend |
| anti-human CD11-PE | 3.9 | BioLegend |
| anti-human CD95-BV421 | DX2 | BioLegend |
| anti-human CXCR5-FITC | J252D4 | BioLegend |
| anti-human IgD-PerCP-Cy5.5 | IA6-2 | BioLegend |
| anti-human FCRL5-APC | 509f6 | BioLegend |
| anti-human T-bet BV711 | 4B10 | BioLegend |
| anti-human IgM BV750 | MHM-88 | BioLegend |
| anti-human CD38 PE-Dazzle594 | HB-7 | BioLegend |
| Viability (Zombie Aqua) |  | BioLegend |
|  |  |  |
| HUMAN IgG ELISA | | |
| **Antibody** | **Clone** | **Manufacturer** |
| Anti-human IgG (capture) | MT91/145 | Mabtech |
| Biotin-conjugated anti-human IgG (detection) | MT78/145 | Mabtech |
|  |  |  |
| B CELL ISOLATION |  |  |
| **Reagent** | **Manufacturer** | **Catalog No.** |
| EasySep^TM^ Human B Cell Isolation Kit | StemCell Technologies | 17954 |
| MojoSort Human anti-PE Nanobeads | BioLegend | 480091 |
|  |  |  |
| CELL STAINING |  |  |
| **Reagent** | **Manufacturer** | **Catalog No.** |
| Human TruStain FcX Fc Receptor Blocking Solution | BioLegend | 422302 |
| Brilliant Stain Buffer | BD Biosciences | 563794 |
| True-Nuclear Transcription Factor Buffer Set | BioLegend | 424401 |
| Cell Staining Buffer | BioLegend | 420201 |
|  |  |  |
| B CELL CULTURE |  |  |
| **Reagent** | **Manufacturer** | **Catalog No.** |
| Recombinant human IL-2 | Peprotech | 200-02-50UG |
| Recombinant human IL-4 | Peprotech | 200-04-20UG |
| Recombinant human IL-21 | Peprotech | 200-21-10UG |
| Recombinant human BAFF | Peprotech | 310-1-20UG |
| Invivogen Ultrapure LPS (TLR4 agonist, E. coli 055:B5) | Invivogen | TLRL-B5LPS |
