# Supplementary figures and images for: Subtype-specific atypical B cell profiles in myasthenia gravis reveal distinct immunopathological pathways
Source: Front Immunol. 2025 Jun 18;16:1608160. doi: 10.3389/fimmu.2025.1608160 (PMC12213399; doi:10.3389/fimmu.2025.1608160)

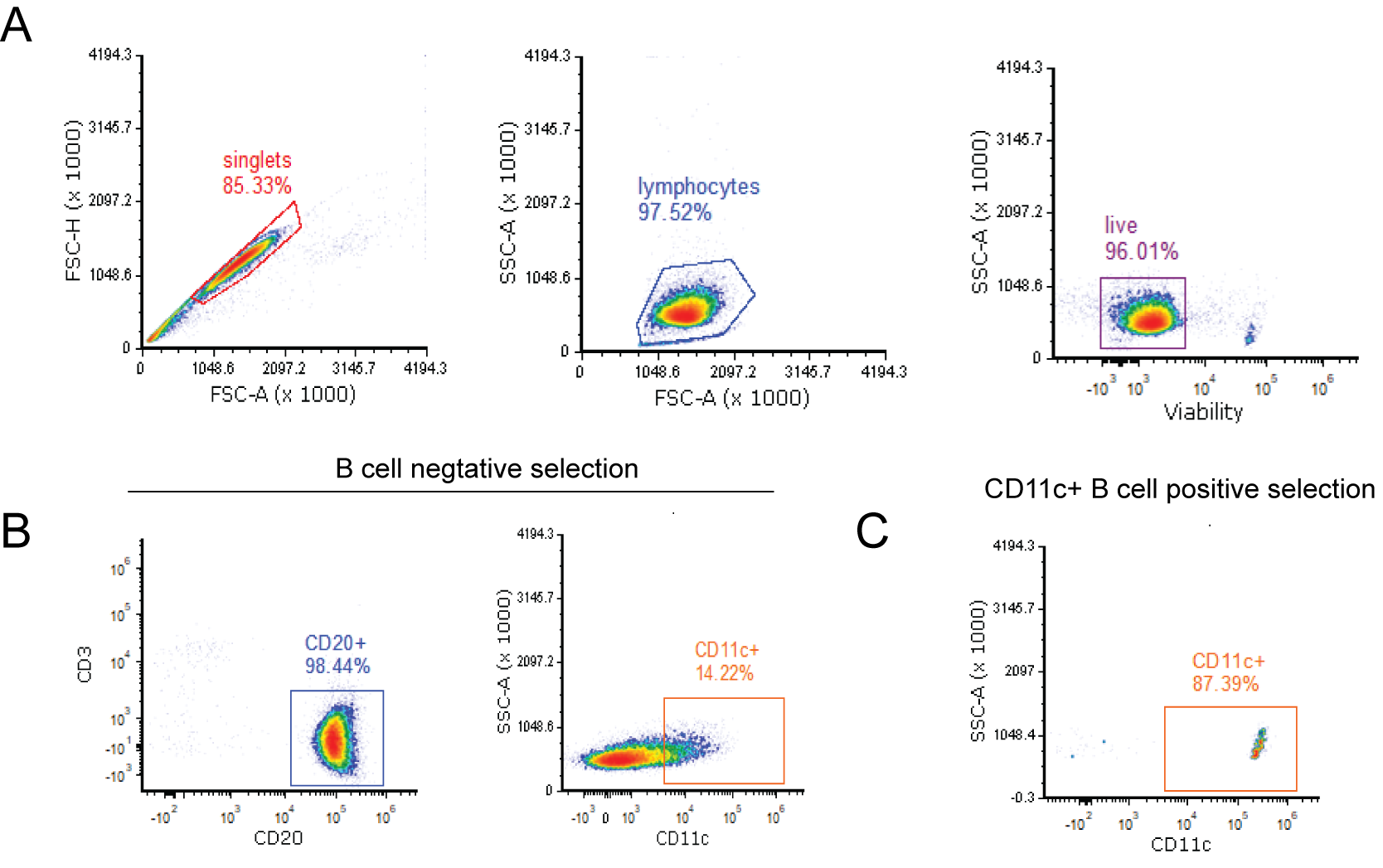

Supplement: Supplementary file 3 [file Image1.tif]

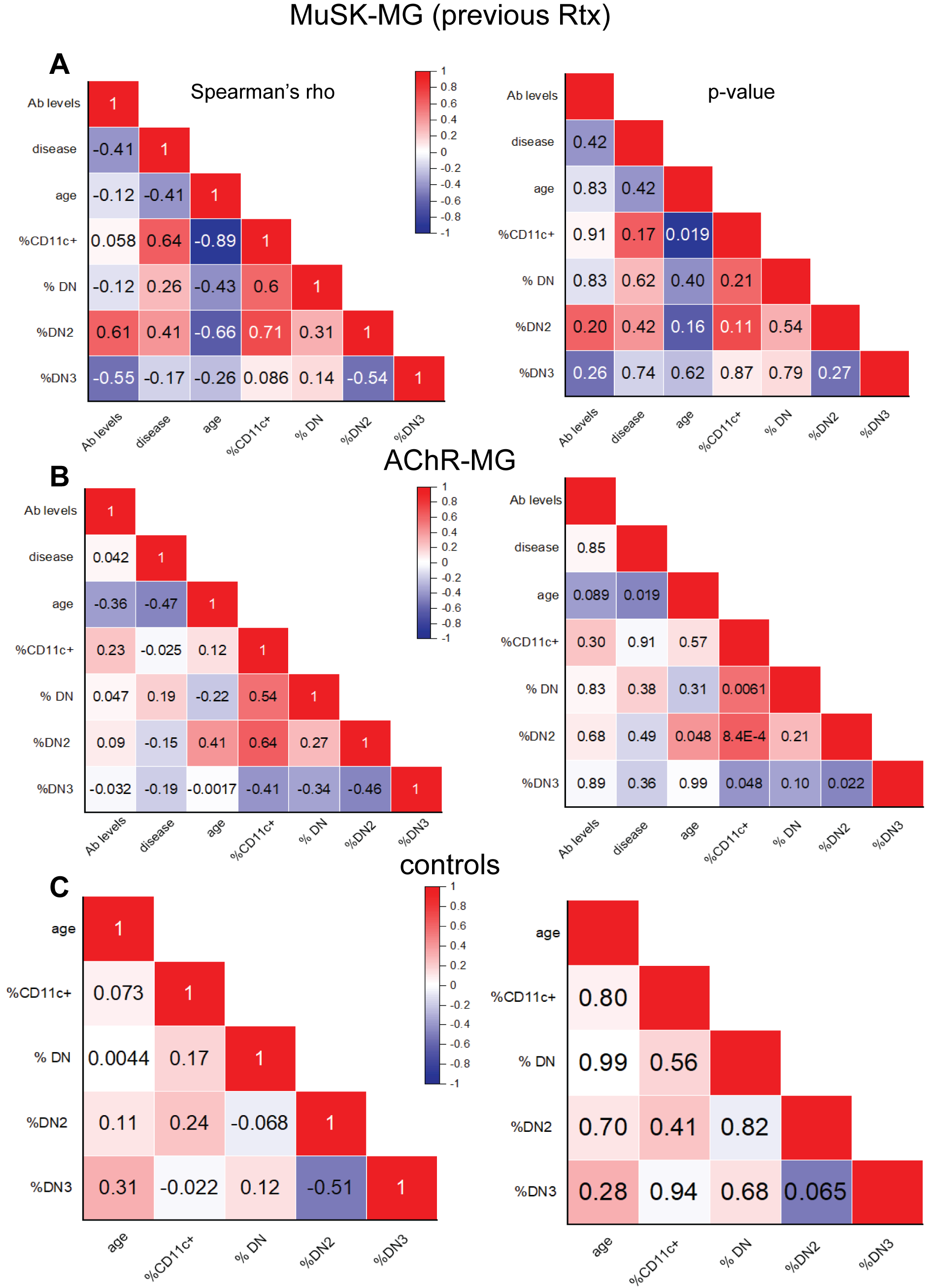

Supplement: Supplementary file 4 [file Image2.tif]

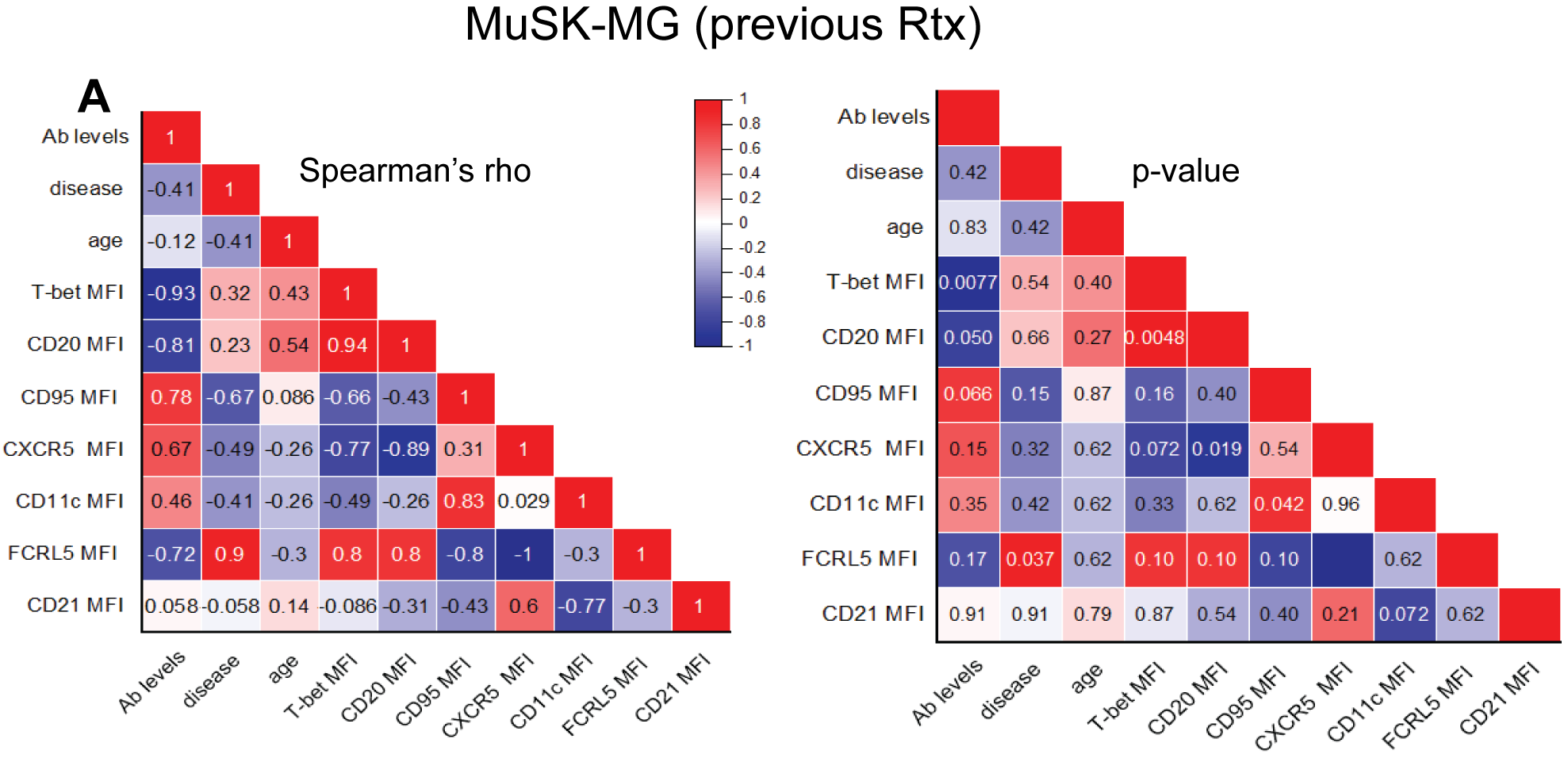

Supplement: Supplementary file 5 [file Image3.tif]

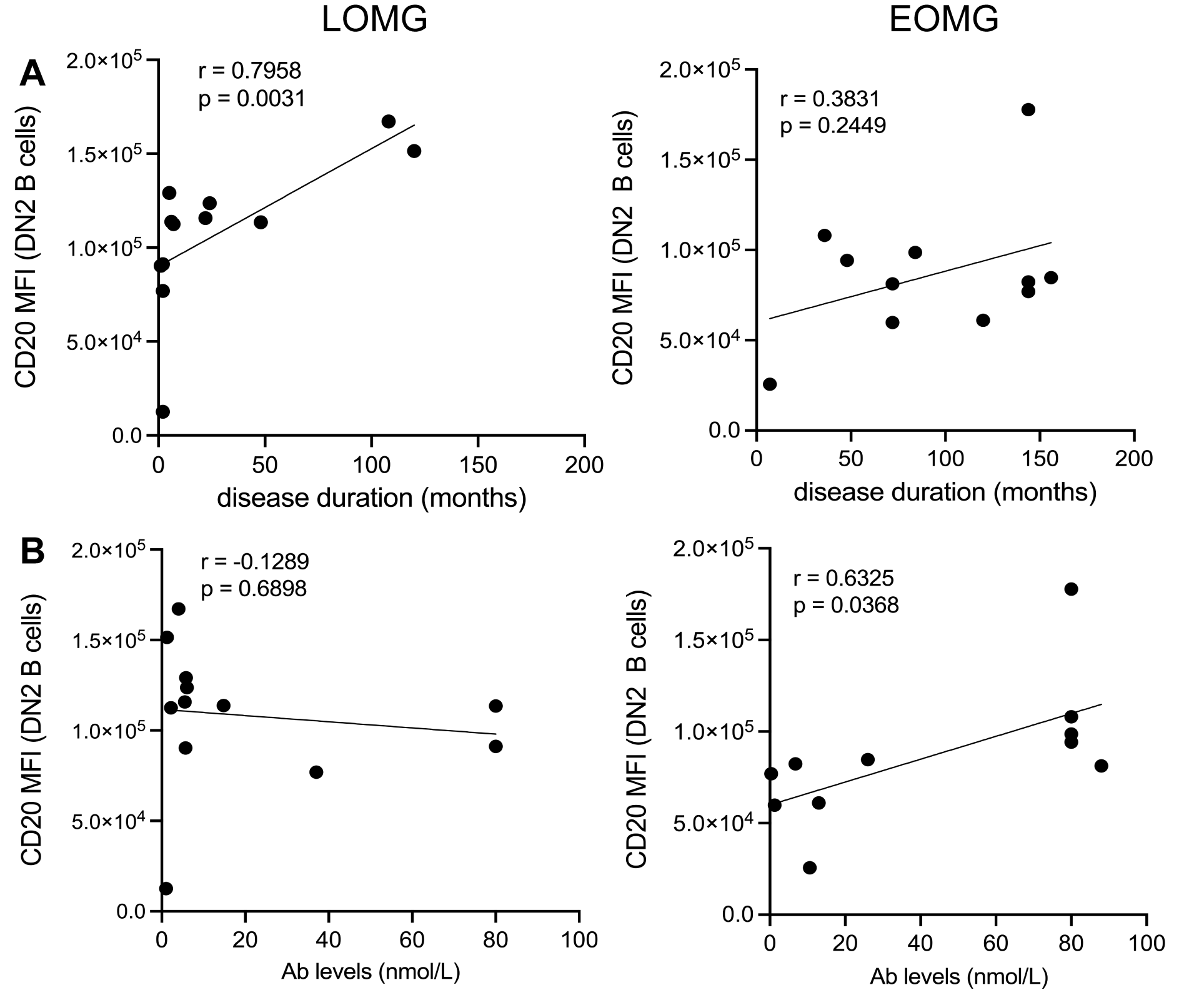

Supplement: Supplementary file 6 [file Image4.tif]
